# USING MDS TO MEASURE TRANSFER TRAUMA IN NURSING HOME RESIDENTS

**DOI:** 10.1093/geroni/igac059.2851

**Published:** 2022-12-20

**Authors:** Ana Montoya, Pil Park, Chiang-Hua Chang, Julie Bynum

**Affiliations:** University of Michigan, Ann Arbor, Michigan, United States; University of Michigan, Ann Arbor, Michigan, United States; University of Michigan, Ann Arbor, Michigan, United States; University of Michigan, Ann Arbor, Michigan, United States

## Abstract

Transferring long-term nursing home residents between facilities has been shown to compromise their quality of life. The negative consequences associated with transfers have been termed transfer trauma and its spectrum spans from functional, cognitive, and emotional decline to hospitalizations, and even death. The objective of this study was to develop a composite measure of transfer trauma after a nursing home-to-nursing home transfer using clinically relevant events and sub-measures covering functional, cognitive, behavioral, and emotional domains based on validated scales available in the Minimum Data Set (MDS) assessments. MDS Data were used to identify long-stay nursing home residents and obtain information on transfers, clinical assessments, and facilities. This study is a cross-sectional cohort analysis of long-stay nursing home residents in the state of Michigan who had a nursing home-to-nursing home transfer during our study period (March 1, 2018-October 31, 2018). We developed a measure of transfer trauma as a composite from 5 previously validated measures: 1) Activity of Daily Living (ADL) Self-Performance Scale; 2) Cognitive Function Scale (CFS); 3) Patient Health Questionnaire-9 (PHQ-9/PHQ-9-OV); 4) Agitated Reactive Behavior Scale (ARBS); 5) falls. The distribution and frequency of each sub-measure were analyzed to assess scaling of the composite measure. Of a total of 817 residents who transferred in our study period, 549 had complete composite measures, and from those 242 (44%) met criteria for transfer trauma. These findings demonstrate the need for further research to identify underlying transfer trauma and develop interventions to ameliorate the negative effects of transfers on this vulnerable population.

